# Characterization and management of long runs of homozygosity in parental nucleus lines and their associated crossbred progeny

**DOI:** 10.1186/s12711-016-0269-y

**Published:** 2016-11-24

**Authors:** Jeremy T. Howard, Francesco Tiezzi, Yijian Huang, Kent A. Gray, Christian Maltecca

**Affiliations:** 1 Department of Animal Science, North Carolina State University, Raleigh, NC 27695-7627 USA; 2 Smithfield Premium Genetics, Rose Hill, NC 28458 USA; 3 Genetics Program, North Carolina State University, Raleigh, NC 27695-7627 USA

## Abstract

**Background:**

In nucleus populations, regions of the genome that have a high frequency of runs of homozygosity (ROH) occur and are associated with a reduction in genetic diversity, as well as adverse effects on fitness. It is currently unclear whether, and to what extent, ROH stretches persist in the crossbred genome and how genomic management in the nucleus population might impact low diversity regions and its implications on the crossbred genome.

**Methods:**

We calculated a ROH statistic based on lengths of 5 (ROH5) or 10 (ROH10) Mb across the genome for genotyped Landrace (LA), Large White (LW) and Duroc (DU) dams. We simulated crossbred dam (LA × LW) and market [DU × (LA × LW)] animal genotypes based on observed parental genotypes and the ROH frequency was tabulated. We conducted a simulation using observed genotypes to determine the impact of minimizing parental relationships on multiple diversity metrics within nucleus herds, i.e. pedigree-(**A**), SNP-by-SNP relationship matrix or ROH relationship matrix. Genome-wide metrics included, pedigree inbreeding, heterozygosity and proportion of the genome in ROH of at least 5 Mb. Lastly, the genome was split into bins of increasing ROH5 frequency and, within each bin, heterozygosity, ROH5 and length (Mb) of ROH were evaluated.

**Results:**

We detected regions showing high frequencies of either ROH5 and/or ROH10 across both LW and LA on SSC1, SSC4, and SSC14, and across all breeds on SSC9. Long haplotypes were shared across parental breeds and thus, regions of ROH persisted in crossbred animals. Averaged across replicates and breeds, progeny had higher levels of heterozygosity (0.0056 ± 0.002%) and lower proportion of the genome in a ROH of at least 5 Mb (−0.015 ± 0.003%) than their parental genomes when genomic relationships were constrained, while pedigree relationships resulted in negligible differences at the genomic level. Across all breeds, only genomic data was able to target low diversity regions.

**Conclusions:**

We show that long stretches of ROH present in the parents persist in crossbred animals. Furthermore, compared to using pedigree relationships, using genomic information to constrain parental relationships resulted in maintaining more genetic diversity and more effectively targeted low diversity regions.

**Electronic supplementary material:**

The online version of this article (doi:10.1186/s12711-016-0269-y) contains supplementary material, which is available to authorized users.

## Background

Swine breeding systems are based on selection within nucleus lines to improve crossbred performance [1]. Crossbreeding programs aim at exploiting both between-breed complementarity of additive genetic effects and heterosis caused by non-additive genetic effects [2]. Furthermore, crossbreeding can result in removal of inbreeding depression that may have accumulated within individual parental lines. Since the advent of dense genotyping platforms, novel selection strategies have been investigated to ensure that crossbred performance is maximized by applying genomic selection [3–7]. In addition to selecting purebred animals for maximum crossbred performance, genomic information on the parental breeds can be used to manage genetic diversity within the parental populations, and also within the genomes of the associated crossbred dam and market animals. Prior to the availability of dense genotype information, breeds were assumed to be unrelated because pedigree information prior to breed formation was not available. With genomic information, it is possible to better understand how frequent long haplotypes are shared across the parental breeds in a swine breeding system. Previous work by Zanella et al. [8] showed that haplotypes are shared across Large White (LW) and Landrace (LA) breeds based on a 50-SNP (single nucleotide polymorphism) run of homozygosity (ROH) metric. A ROH is generated when an individual receives a haplotype that is identical by descent from each parent [9]. Parents can pass on identical chromosomal segments to an offspring even when the relationship between them is very distant, which creates a continuum of homozygous segment length [10, 11]. It is currently unclear how frequently ROH persist in a crossbred population and whether longer ROH than 5 or 10 Mb exist.

Traditionally, ROH metrics have been used as a measure to detect regions of the genome that have undergone positive selection [12–16]. Signatures of selection are characterized by distributions of nucleotides around favorable mutations at frequencies that differ statistically from that expected purely by chance due to directional selection, which increases the frequency of the favorable allele over time [17]. Nucleotides that are linked to the favorable mutation also tend to increase in frequency, a phenomenon referred to as “hitchhiking” [18] and simulation studies have shown that this occurs during selection [19]. Therefore, the use of a ROH metric can provide clues about which regions of the genome have undergone directional selection and how these regions differ between breeds that were selected for different objectives [i.e. terminal (i.e. Duroc) vs. maternal (i.e. Landrace and Large White)]. Genomic regions with high levels of ROH have a reduced level of genetic diversity and a higher level of homozygosity compared to the rest of the genome [20]. Also, previous research showed that long ROH are enriched with deleterious variants compared to regions that are not within in a ROH [21, 22].

The genetic diversity of a swine population can be managed at the population or animal level. Previous research has been conducted at the population level, with the aim of restricting the rate of pedigree or genomic inbreeding to a desired level, while maximizing the long-term genetic gain [23–27]. The approach at the animal level is based on minimizing homozygosity and maximizing haplotypic diversity in the next generation based on mate allocation. The criteria for choosing mating pairs was primarily determined by using a relationship matrix [28–31], which allows mating between the least related individuals, which in turn minimizes the homozygosity in the next generation [28]. Relationship matrices can be constructed by using information on pedigree relationships (**A**) [32] or based on a SNP-by-SNP relationship matrix (**SNPRM**) [33, 34], or methods based on a ROH relationship matrix (**ROHRM**) [30, 35]. A **SNPRM** assumes that SNPs are unlinked and therefore does not fully account for the fact that SNPs that are located on the same homologous chromosomes are inherited together unless a recombination event occurs between them [36].

The diversity of a breeding population at the nucleus level has a strong impact on the capacity of the population to (1) attain maximum performance across a variety of environments, (2) sustain genetic improvement for traits of economic importance and (3) allow for rapid changes in the breeding objective when faced with changes in the economics that drive the production system [37, 38]. Using genomic information within the parental lines [39] allows management of genomic diversity in the crossbred progeny even in the absence of genotypic data on the crossbred animals. In addition, in the purebred lines, regions with a high frequency of ROH are most susceptible to a loss of diversity due to selection and therefore are the most critical regions in which genetic diversity needs to be maintained in the parental populations. Lastly, at the crossbred level, the persistence of ROH could potentially limit the expression of both breed complementarity and heterosis. Here, we hypothesize that the **ROHRM** can be effectively used in mating plans and potentially allows for specific regions of the genome with a high frequency of ROH to be “targeted” in order to reduce the frequency and length of ROH more effectively than either SNP-by-SNP or pedigree-based relationship matrices. Therefore, the objectives of our study were: (1) to characterize the ROH frequency using observed genotypes across three parental breeds (Landrace (LA), Large White (LW) and Duroc (DU)) and its relationship with the ROH frequency in the simulated genomes of the crossbred dams (LA × LW) and market animals [DU × (LA × LW)]; and (2) to determine the impact of using different relationships to minimize parental relationships in mating plans within the three purebred lines based on heterozygosity and frequency and length of the ROH and their implications for the crossbred genome.

## Methods

### Animals and genotypes

No animal care approval was required for this work since all genotypes and records came from data that were available from previous studies. Genotypic data from multiple commercial purebred nucleus selection lines, including DU (n = 2050), LA (n = 1225) and LW (n = 1440), were obtained from Smithfield Premium Genetics (Rose Hill, NC) and were derived from the Illumina PorcineSNP60K BeadChip (Illumina Inc., San Diego) and the GGP-Porcine chip that includes about 10,000 SNPs (GeneSeek Inc., a Neogen Co., Lincoln). Prior to the imputation of missing genotypes and from low-density to medium-density, multiple quality control edits were conducted, including the removal of individuals and SNPs with call rates lower than 0.90, SNPs with a minor allele frequency (MAF) lower than 0.002, and a p value of a Chi square test for Hardy–Weinberg equilibrium lower than 0.0001. Using a larger set of genotyped individuals within the DU (n = 8705), LA (n = 5530) and LW (n = 7201) populations, imputation and determination of the genotype phase were conducted using Beagle (Version 3) [40] within each breed separately. SNPs that had an imputation accuracy lower than 0.90, SNPs that were not mapped to swine genome build 10.2 and SNPs on sex chromosomes were also excluded. The map file used was based on version 2 of the Illumina PorcineSNP60K BeadChip genotype platform and SNPs that were not shared across genotype platforms were removed. To minimize time-related bias that could result from selection that occurred within each line and to compare populations as equitably as possible, only animals born in 2012 were used, as previously done by Howard et al. [41]. Furthermore, due to the comparably small number of sires, only females were used to characterize differences in homozygosity, which resulted in the use of 1144 LA, 1341 LW and 1512 DU females. Numbers of animals per genotype platform and of SNPs after quality control for each breed are in Table 1.

**Table 1:** Summary of numbers of animals^a^ and SNPs after quality control by breed

Breed	After quality control					
	Animals	Females 2012	Males 2012	SNP	SNP ROH5^c^	SNP ROH10^d^
DU	2050	1512	538	34,904	34,179	34,181
LA	1225	1144	81	41,489	41,272	41,331
LW	1440	1341	99	39,671	39,488	39,501
LA × LW^b^	8100	–	–	35,191	35,059	35,054
DU × (LA × LW)^b^	53,900	–	–	26,548	25,559	25,490

*DU* Duroc, *LW* Large White, *LA* Landrace

^a^The females born in 2012 were used in the principal component analysis and to characterize the ROH frequency within the purebred population, while both males and females born in 2012 were used to generate the crossbred genome to characterize ROH frequency and in mating designs

^b^Genotypes were simulated based on the purebred genotypes and therefore were not genotyped on a platform

^c^Refers to the number of SNPs used in the calculation to determine whether a given SNP was in a ROH of at least 5 Mb

^d^Refers to the number of SNPs used in the calculation to determine whether a given SNP was in a ROH of at least 10 Mb

### Population differentiation

To characterize the degree of genome-wide population differentiation that exists between the three populations, we calculated Wright’s *F*_st_ statistic and performed a principal component analysis (PCA) on **SNPRM**. Wright’s *F*_st_ was obtained as outlined in Weir and Cockerham [42]. The **SNPRM** were constructed based on genome-wide SNPs that were in common across the three populations after quality control (n = 26,510 SNPs) following the method outlined by Yang et al. [33]. Briefly, the **SNPRM** between individual *i* and individual *j* across *m* SNPs was calculated using the following formula:

$$\begin{aligned} SNPRMij & = \frac{1}{N}\mathop \sum \limits_{m} \frac{{(x_{mj} - 2*0.5)(x_{mi} - 2*0.5)}}{{2*0.5*\left( {1 - 0.5} \right)}}\quad {\text{if}}\; j \ne i, \\ SNPRMij & = 1 + \frac{1}{N}\mathop \sum \limits_{m} \frac{{x_{mj}^{2} - \left( {1 + 2*0.5} \right)x_{mj} + 2*0.5^{2} }}{{2*0.5*\left( {1 - 0.5} \right)}}\quad{\text{if}}\; j = i, \\ \end{aligned}$$

where *N* is the number of SNPs and *x*_*m*_ is the genotype at SNP_*m*_. Genotypes were coded as 0 for the homozygote, 2 for the other homozygote and 1 for the heterozygote. The frequency of 0.5 was used instead of the observed frequency, as in Yang et al. [33], due to allele frequencies differing between the populations and the potential for **SNPRM** to be greatly impacted by re-weighting relationships based on common versus rare alleles in a multiple breed relationship matrix. A PCA was conducted on the **SNPRM** using the R function *eigen* [43]. The first two principal components were plotted to determine the degree of genetic differentiation across the populations. The percentage of variance explained by the first principal component was estimated by dividing the variance explained by the first principal component over the total variance.

### Characterizing runs of homozygosity across and within populations

The distribution and frequency of long stretches of homozygosity were investigated using the method outlined by Kim et al. [44] based on ROH cutoff lengths of 5 (ROH5) and 10 (ROH10) Mb. Briefly, for a given ROH Mb length cutoff, a sliding window approach was used to define ROH regions and a ROH was declared for an individual when the region contained only contiguous homozygous SNP genotypes (no heterozygous SNP genotypes observed). The sliding window approach started with the first SNP on a chromosome and combined all SNPs within a set cutoff length into a window. Then, the ROH status was declared and the window was shifted by one SNP to form a new window that had a length greater than the cutoff length. This process was repeated until the end of the chromosome. After the ROH statuses of all windows were declared, the ROH5 or ROH10 status of a SNP was defined based on whether it belonged to a ROH of at least 5 or 10 Mb, respectively. The ROH5 and ROH10 statuses of a SNP were tagged as 1 if the SNP was in a ROH and 0 otherwise. If a SNP was in a ROH of at least 10 Mb, it was by default in a ROH of at least 5 Mb, resulting in some degree of nesting for the two ROH measures. In this study, we considered ROH lengths of 5 and 10 Mb to characterize medium and long stretches of homozygosity. The minimum cutoff was set at 5 Mb since cutoffs that are less than 5 Mb have a higher likelihood that the ROH contains a small number of SNPs due to their uneven distribution. In addition, ROH with a small number of SNPs were removed from the analysis by removing any ROH window that had a SNP count that was less than 2 standard deviations from the average number of SNPs contained within a ROH window within each breed. Total numbers of ROH5 and ROH10 SNPs used within each breed are in Table 1. After quality control, the average (±SD) number of SNPs in a window of 5 Mb for the DU, LA and LW breeds was 92.3 (±24.5), 95.5 (±24.2) and 92.3 (±24.5), respectively. Within 10-Mb windows, the average (±SD) number of SNPs was 158.3 (±39.6), 185.6 (±42.0) and 178.6 (±42.1), for the DU, LA and LW breeds, respectively. For each individual, the proportion of the genome that was included within a ROH was estimated as the sum of ROH lengths (Mb) of an individual divided by the total Mb length across all 18 pig autosomes.

For a given ROH cutoff length, regions of the genome with a high ROH5 or ROH10 frequency were declared using a two-stage approach. The first stage involved isolating regions with a high frequency of ROH by keeping the top 5% SNPs based on ROH5 or ROH10 frequency. The second stage involved aggregating individual SNPs that formed a contiguous set into a single region and calculating the number of SNPs within that region. Lastly, regions that had a SNP count less than 2 standard deviations from the average number of SNPs included in a ROH window for the given breed were removed. In this approach, the second stage was necessary because ROH5 and ROH10 are a function of genomic regions of 5 or 10 Mb, respectively, and therefore it is expected that regions with high ROH5 or ROH10 frequencies should be maintained within a contiguous set of SNP. Differences in ROH frequencies between populations were also characterized based on pairwise ROH5 or ROH10 comparisons for SNPs that were in common across populations. In this case, ROH regions were defined by using the complete set of SNPs within each population to allow for a larger number of SNPs when calculating the ROH5 or ROH10 metric within a breed. The same two-stage approach as above was also used to identify the presence of different ROH5 and ROH10 regions between two breeds. Regions that displayed consistent differences between maternal (LW and LA) and terminal lines (DU) or high frequencies of either ROH5 or ROH10 across multiple breeds were further investigated to identify regions that potentially had long stretches of shared haplotypes based on a high frequency of ROH5 and/or ROH10 across multiple breeds. To further analyze the regions that displayed high frequencies of ROH5 or ROH10 across multiple populations, regions that covered 500 kb before and after the SNP with the highest ROH5 or ROH10 frequency within each region were investigated by using AnimalQTLdb [45].

### Persistence of runs of homozygosity in maternal crossbred and commercial crossbred animals

To assess the percentage of ROH that were consistent in the crossbred dams and market animals due to a shared ROH between the parents used in the cross, crossbred animal genotypes were simulated based on the observed sire and dam genotypes. We had to use a simulation because of the lack of observed crossbred genotypes, and because two parental lines that each have a high ROH frequency across the same region may not have the same ROH genotype and therefore ROH may not persist in the associated crossbred genome. Crossbred animals were generated based on a traditional commercial swine breeding system. The crossbred dam was simulated by mating LA males to LW females, while market animals were created by mating DU males to the simulated crossbred dams. Genotypes from animals born in 2012 were used as parents for each simulation, as shown in Table 1. Gametes were created based on phased genotypes, with a crossover probability that was simulated from a Poisson distribution and based on the length of the chromosome in Morgans. Crossover locations were sampled at random from a uniform distribution. The length in Morgans for each chromosome was set following Rohrer et al. [46]. Each male was mated to a random set of 100 females. For each mating pair, a paternal and a maternal gamete were generated and the resulting genotype was used to calculate the ROH5 and ROH10 status of each SNP, as outlined previously. The average (±SD) number of SNPs within a 5-Mb ROH window for the crossbred dams and market animals was 82.9 (±23.8) and 70.5 (±18.1), respectively. Similarly, the average (±SD) number of SNPs for 10-Mb windows was 160.4 (±23.8) and 133.4 (±30.4) for the same crossbred populations, respectively. Total numbers of ROH5 and ROH10 SNPs for the crossbred dams and crossbred market animals are in Table 1. For each simulated crossbred individual, the proportion of the genome that was included within a ROH for an individual was estimated as the sum of its ROH lengths (Mb) divided by the total Mb length across all 18 porcine autosomes. The same two-stage approach that was discussed above was also used to identify the presence of high frequency ROH5 and ROH10 SNP regions in the crossbred dams and market animals. The only difference was that in the second stage, regions that had a SNP count less than 2 and 1 standard deviations from the average number of SNPs within a ROH window for the crossbred dams and market animals, respectively, were included in the analysis. A more stringent threshold on the SNP count within an ROH was set for the crossbred market animals since the number of SNPs in common across the three populations was smaller and therefore the presence of a small number of SNPs within a ROH increased as the number of SNPs decreased.

### Mating designs to minimize long stretches of homozygosity within nucleus populations

To better understand how mate allocation strategies that use different relationship matrices might impact the frequency of homozygous stretches and their associated lengths, mating designs aimed at minimizing relationships based on **A**, **SNPRM** or **ROHRM** were simulated. Matrix **A** was constructed based on the recursive algorithm of Henderson [32] and was traced back until all ancestors were unknown. The number of generations that was traced back was 12 for LA and LW and 11 for DU. The **SNPRM** was constructed within each breed using the method as described by Yang et al. [33] and as outlined previously in the section on population differentiation. The **ROHRM** was created based on modifications of methods used by Pryce et al. [30] and Hickey et al. [35]. The concept behind the **ROHRM** is that a haplotype that is shared between the parents results in a potential ROH in the progeny, which can occur even if a ROH is not observed in the two parental genomes. Using a ROH cutoff length of 5 Mb, one-SNP sliding windows were generated across the genome. For each window *k*, the **ROHRM**_*k*_ was computed as the number of haplotypes that were exactly the same (i.e. result in a ROH) for individuals *i* and *j* divided by 2. The minimum and maximum numbers of haplotypes that can be the same is 0 and 4, respectively. The **ROHRM**_*k*_ for a given window, therefore, is essentially the ROH-based version of the classical gametic relationship between a pair of individuals [47]. After calculating all **ROHRM**_*k*_ across all windows, a genome-wide ROHRM was generated as the average of all **ROHRM**_*k*_ matrices. An example of how **ROHRM**_*k*_ was constructed along with the prototype C++ code, an example genotype file and an example map file are in Additional files 1, 2 and 3, respectively.

Using the previously described relationship matrices, mating designs were constructed by mimicking the size of an idealized nucleus population of ~625 females. Within each replicate, 25 sires and 625 dams were randomly chosen from the full set as potential parents. Matings were replicated 50 times. Within each replicate, mates were selected based on sequential selection of least-related (SSLR) mates, as outlined by Pryce et al. [30] or by random mating. The relationship matrices used in SSLR were either **A**, **SNPRM**, or **ROHRM** within each breed. The SSLR algorithm was implemented by constructing a vector (sire count; SC), initialized to 0, which kept track of the total number of times that a sire was assigned a mating pair. The maximum number of selected mates for a given sire was set at 25 for all populations. The algorithm proceeded across all dams and, for each dam, it identified the sire that was least related to it based on a given relationship metric. Once a sire was found, its number of mates was determined and if it was not at its maximum mate number, the cell in the SC vector pertaining to the sire was incremented by 1. If the sire was at its maximum mate number, then the next least related sire was chosen, its number of mates was determined, etc., and the process was repeated until a sire was not at its maximum number of mates. Once the last dam and sire combination was determined, the SC vector had a value of 25 for all sires. For each sire-dam combination, one progeny was simulated using the same methodology as used above to investigate the persistence of ROH in the crossbred genomes. To investigate the impact of different relationship matrices at the genome-wide level, multiple metrics were computed, including pedigree-based inbreeding, marker heterozygosity, and the proportion of the genome in a ROH. To investigate the impact of different relationship matrices on reducing the length and frequencies of long stretches of ROH for regions with low levels of genetic diversity, the genome was split into quantiles of increasing ROH5 frequency for a SNP. In order to make quantile cutoff values (i.e. the ROH5 frequency for a SNP) that were consistent within replicates across scenarios, the cutoff within each replicate was based on the ROH5 in the subset of parents that were sampled for that replicate. The quantiles were binned into four classes based on percentiles, i.e. [0, 49.99], [50, 74.99], [75, 89.99] and [90, 100]. For each scenario, multiple parameters were computed for each quantile, i.e. mean heterozygosity, mean ROH5 frequency, and mean ROH length (Mb) for SNPs that were contained within an ROH of at least 5 Mb. All statistics were expressed as the average difference between the progeny generation and the parental generation across replicates and therefore represent the increase or decrease in diversity of the progeny genome compared to the parental genome. A C++ program that reads in male and female animals with genotypes and a marker map and outputs simulated progeny genotypes, and all the associated statistics are available upon request.

## Results

### Genetic characterization of purebred and crossbred populations

**Fig. 1:**
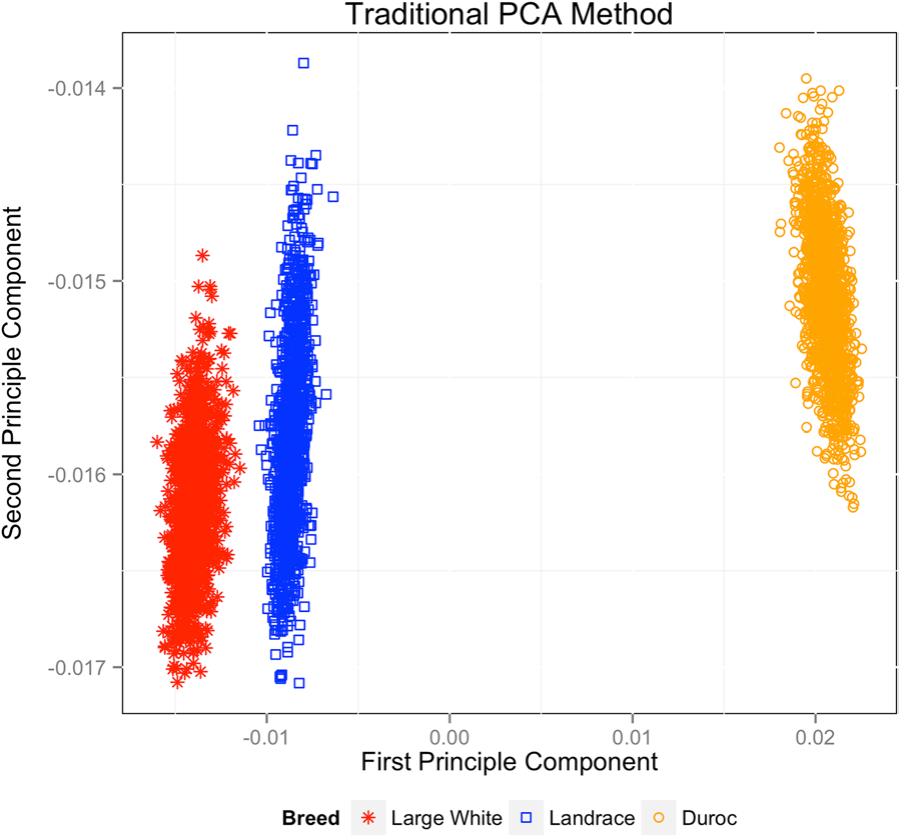
First versus second principal components based on the genomic relationship matrix for Duroc, Large White, and Landrace

Figure 1 shows a scatterplot of the first (PC1) versus the second principal component (PC2) of the SNPRM, The variance explained by the PC1 and PC2 were 25.0 and 16.2%, respectively, and resulted in clear divergence between the three breeds. The mean (±SD) *F*_ST_ statistic for LW versus LA, LW versus DU, and LA versus DU were equal to 0.115 (0.138), 0.152 (0.172) and 0.145 (0.172), respectively. Thus, based on traditional metrics calculated on averages across the genome, the breeds appeared to be substantially different at the genome-wide level.

**Fig. 2:**
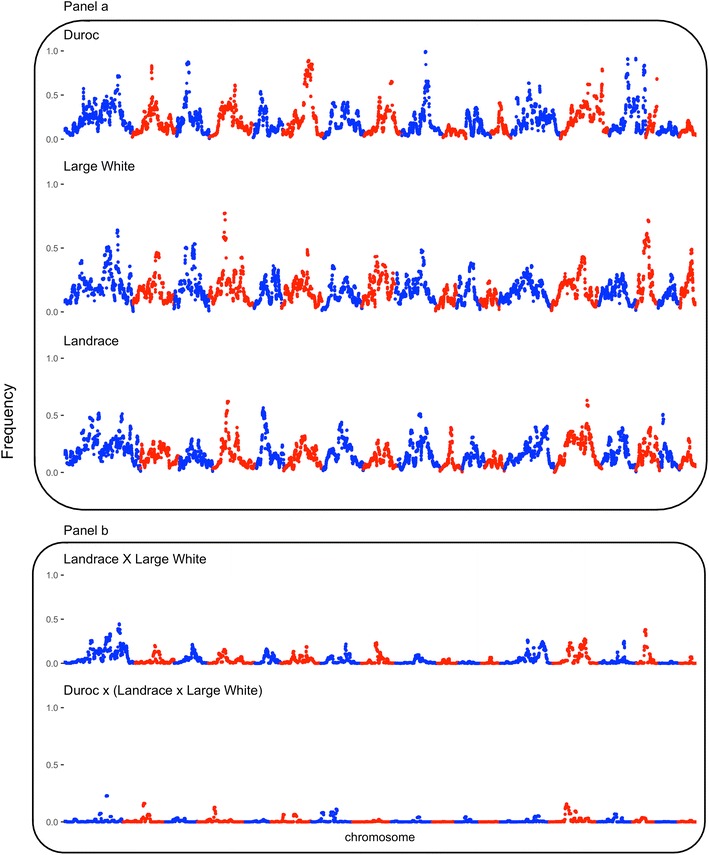
Frequency of a SNP being in a ROH of 5 Mb across the genome for purebred (**a**) and crossbred populations (**b**)

**Fig. 3:**
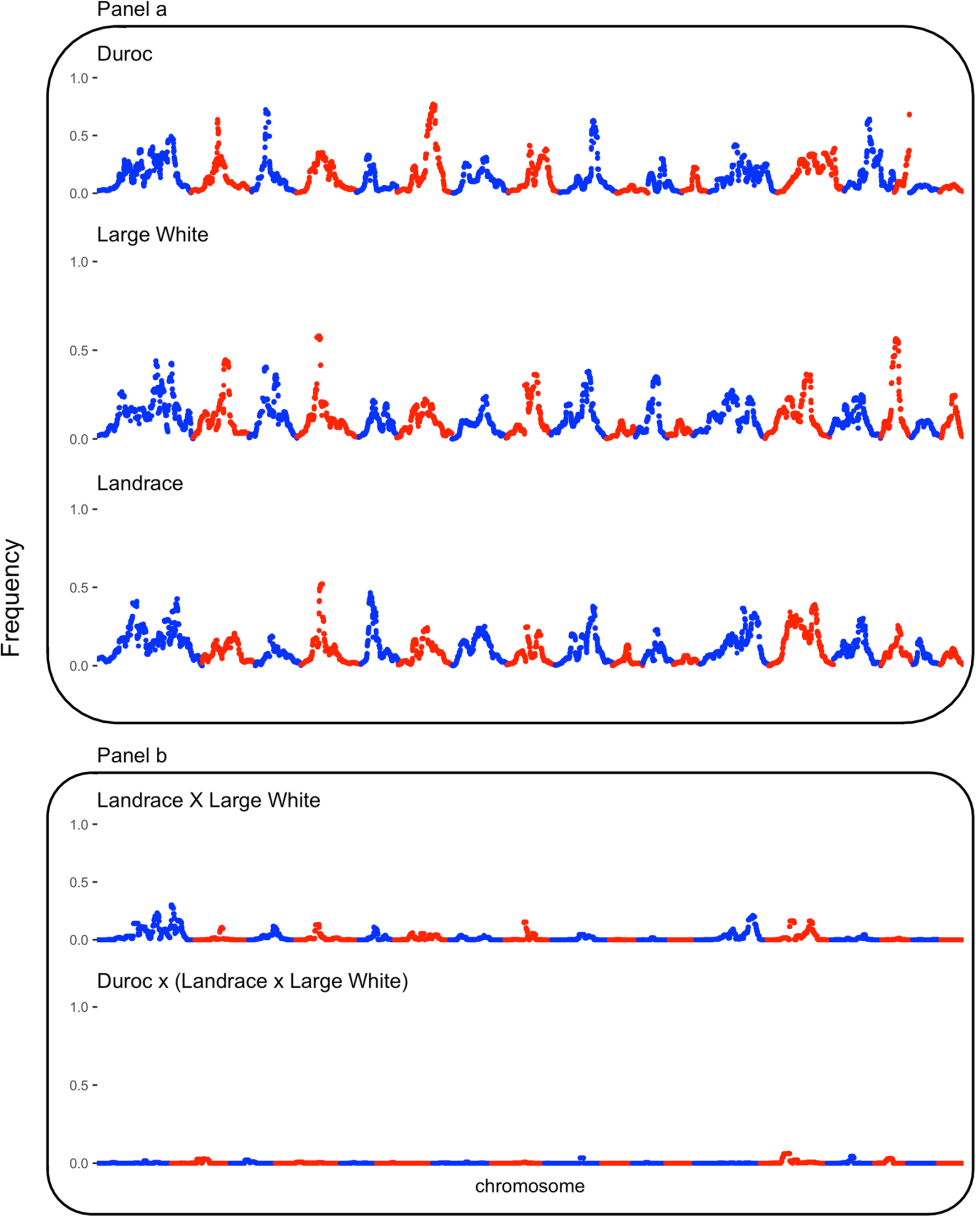
Frequency of a SNP being in a ROH of 10 Mb across the genome for purebred (**a**) and crossbred populations (**b**)

Figures 2 and 3 show the ROH5 and ROH10 frequency, respectively, across the genome for the three purebred populations in panel (a) and for the two crossbred populations in panel (b). As shown in panel (a), the purebred breeds display some degree of similarity across the genome with regard to regions that had high frequencies of ROH, although there were also regions that had high frequencies of ROH that were breed-specific. The average (±SD) proportion of the genome in a ROH of at least 5 Mb (LA: 0.17 ± 0.04; LW: 0.19 ± 0.04; DU: 0.20 ± 0.04) and 10 Mb (LA: 0.11 ± 0.04; LW: 0.13 ± 0.04; DU: 0.13 ± 0.04) was similar across the three purebred breeds. This is in agreement with pedigree-based inbreeding coefficients (LA: 1.04 ± 0.02; LW: 0.05 ± 0.02; DU: 1.03 ± 0.02) and the diagonals of the **SNPRM** (LA: 1.35 ± 0.04; LW: 1.34 ± 0.04; DU: 1.35 ± 0.03), which did not show large differences between populations. However, numerically there was some re-ranking between populations in the mean inbreeding level, depending on the inbreeding metric used. Regions of the genome with high frequencies of ROH5 or ROH10 across multiple purebred and/or crossbred genomes are in Table 2. Regions of high frequencies of either ROH5 and/or ROH10 across the two maternal breeds were detected on SSC1 (227.0–247.1 Mb), SSC4 (42.1–61.3 Mb) and SSC14 (98.0–111.7 Mb). Furthermore, a region on SSC9 (72.6–104.3 Mb) was found to be in the top 5% for either or both ROH5 and ROH10 across all three breeds. A region on SSC3 displayed high levels of autozygosity across both DU and LW breeds. Differences between ROH5 and ROH10 frequencies between the purebred breeds are in Table 3. Differences in ROH5 and ROH10 frequencies between the terminal breed and both maternal breeds were found on SSC1 (248.7–264.2 Mb), SSC3 (36.4–59.5 Mb), SSC6 (82.3–119.6 Mb) and SSC14 (121.0–132.5 Mb). Across all four regions, frequencies of both ROH5 and ROH10 were higher in the DU breed than in both maternal breeds. Within regions, multiple QTL have been detected based on the AnimalQTLdb [45], which are listed in Additional file 4.

**Table 2:** Regions of the genome with high levels of autozygosity across multiple breed groups

SSC	Location^a^ (Mb)	DU		LW		LA		LA × LW		DU × (LA × LW)	
		5 Mb^b^	10 Mb^c^	5 Mb^b^	10 Mb^c^	5 Mb^b^	10 Mb^c^	5 Mb^b^	10 Mb^c^	5 Mb^b^	10 Mb^c^
1	76.1–88.2					76.1|0.48	88.1|0.40	82.0|0.26	88.1|0.14		
1	134.0–162.6			153.6|0.51				159.5|0.30	151.8|0.23		
1	172.7–198.2			177.8|0.51				178.3|0.33		194.2|0.07	
1	227.0–247.1			241.9|0.64	241.9|0.43	237.9|0.50	234.4|0.42	239.3|0.45	232.2|0.30		
1	248.9–268.7	256.7|0.72	256.7|0.49					262.1|0.22	261.0|0.17		
2	106.4–118.9			110.7|0.46	110.7|0.45					112.6|0.07	108.4|0.03
3	37.7–59.5	53.6|0.88	48.8|0.72	49.2|0.51	50.2|0.40						55.7|0.02
4	42.1–61.3			53.5|0.77	49.2|0.58	53.0|0.61	48.1|0.52		56.6|0.13	49.9|0.13	
6	82.3–119.1	99.8|0.89	100.0|0.77					101.1|0.19		88.7|0.06	
7	52.8–60.8					58.5|0.43				58.8|0.09	
9	72.6–104.3	99.7|0.66	85.5|0.63	90.8|0.48	95.8|0.38		96.2|0.37				
13	161.1–189.5					189.0|0.40	173.3|0.33	178.2|0.25	178.2|0.21	174.5|0.05	
14	98.0–111.7			103.6|0.43	98.9|0.37	102.7|0.62	108.8|0.39	102.8|0.28	100.2|0.16		
15	60.3–85.6	66.1|0.91	66.1|0.64							62.0|0.07	61.0|0.04

*DU* Duroc, *LW* Large White, *LA* Landrace

^a^Represents regions in base pairs (if regions overlapped across populations or another ROH cutoff length then it was aggregated into a single region)

^b^Refers to location with maximum frequency of ROH based on a 5 Mb cutoff and its associated frequency after vertical line

^c^Location with maximum frequency of ROH based on a 10 Mb cutoff and its associated frequency after vertical line

**Table 3:** Regions of the genome with high levels of autozygosity across the genome for the Duroc, Large White, and Landrace breeds

SSC	Location^a^ (Mb)	LW–DU		LA–DU		LW–LA	
		5 Mb^b^	10 Mb^c^	5 Mb^b^	10 Mb^c^	5 Mb^b^	10 Mb^c^
1	76.1–88.1					88.1|−0.32	88.1|−0.30
1	107.2–115.8					110.5|−0.35	
1	151.2–160.2					156.2|0.31	
1	248.7–264.2	257.6|−0.57	257.9|−0.36	257.6|−0.53	256.7|−0.34		
2	106.4–118.7		110.8|0.34				109.6|0.28
3	36.4–59.5	55.9|−0.68	55.9|−0.51	55.9|−0.67	55.6|−0.57	47.4|0.29	
3	74.1–90.9					77.8|0.40	80.4|0.28
4	48.9–61.0	53.5|0.42				54.2|0.45	
4	108.2–114.2					112|0.36	
5	17.9–34.0					20.1|−0.44	24.3|−0.37
6	82.3–119.6	112.9|−0.72	108.8|−0.64	112.9|−0.73	108.8|−0.65		
9	72.6–95.9	77.8|−0.73		82.6|−0.73	77.6|−0.40		
13	15.0–19.9	19.7|−0.38		15.0|−0.34			
13	56.2–66.3	58.9|−0.51	59.2|−0.32	58.3|−0.49			
14	121.0–132.5	132.4|−0.64	132.4|−0.34	132.4|−0.66	132.4|−0.33		
15	59.4–84.2	66.4|−0.78	66.4|−0.54	67.9|−0.74	69.3|−0.51		
15	144.7–152.4	150.1|−0.78		147.2|−0.78			
16	32.1–52.2					37.4|0.45	37.4|0.43
18	40.8–46.4	43.5|0.35				43.5|0.41	

*DU* Duroc, *LW* Large White, *LA* Landrace

^a^Represents regions in base pairs (if regions overlapped across populations or another ROH cutoff length then it was aggregated into a single region)

^b^Location with maximum ROH frequency based on a 5 Mb cutoff and its associated frequency after vertical line

^c^Location with maximum ROH frequency based on a 10 Mb cutoff and its associated frequency after vertical line

To determine whether ROH persist in crossbred animals as a result of long stretches of shared parental haplotypes, simulated genotypes were generated based on the observed parental genotypes. These results are in panel (b) of Figs. 2 and 3 for ROH5 and ROH10, respectively. For the majority of the genome, the high frequency of ROH in the parental breeds did not occur in the crossbred animals, although some regions of the genome persisted in the crossbred animals due to shared haplotypes between parental breeds. The average (±SD) proportion of the genome in a ROH of at least 5 Mb was 0.05 (±0.02) and 0.008 (±0.005) for the crossbred dams and crossbred market animals, respectively. Similarly, the average (±SD) proportion of the genome in a ROH of at least 10 Mb was 0.02 (±0.01) and 0.002 (±0.003) for the crossbred dams and crossbred market animals, respectively. The three regions that displayed high frequencies of ROH5 and/or ROH10 within the maternal breeds on SSC1, 4 and 14 were also present in the top 5% ROH5 and ROH10 within the crossbred dams. Furthermore and as expected, the two maternal breeds shared a larger number of haplotypes compared to the final crossbred market animals, for which the majority of high-frequency ROH regions disappeared after crossing. Lastly, if a region had a high ROH frequency in any of the breeds, it was more likely to persist in the crossbred progeny.

### Mating designs to minimize long stretches of homozygosity

The correlation of the off-diagonal elements between the three matrices within each breed, using all available sires and dams, is in Table 4. The average correlation across breeds between off-diagonal elements of the **SNPRM** and **ROHRM** was high (0.89), compared to that between the **SNPRM** and **A** (0.59) and the **ROHRM** and **A** (0.71). The impact of constraining pedigree or genomic-based relationships in the parents on genome-wide estimates of inbreeding for the three breeds is in Table 5. Across the three breeds, minimizing relationships based on **A** had a favorable impact on reducing pedigree inbreeding. At the genomic level, **A** resulted in negligible changes in comparison to the changes observed when minimizing relationships based on genomic information. Furthermore, in LW minimizing **A** resulted in a slight unfavorable decrease in genome-wide heterozygosity and an increase in the proportion of the genome in ROH, while for LA and DU minimizing **A** resulted in a slight favorable increase in genome-wide heterozygosity and a decrease in the proportion of the genome in ROH. Thus, minimizing relationships based on matrix **A** had limited impact on the overall diversity at the genomic level based on genome-wide heterozygosity and the proportion of a genome in ROH. Across all breeds, minimizing relationships based on **SNPRM** or **ROHRM** resulted in a similar increase in heterozygosity and a similar decrease in the proportion of the genome in ROH. Compared to the parental genome, the average (±SD) heterozygosity in the progeny using genomic data (i.e. **SNPRM** or **ROHRM**) increased by 0.0056% (±0.002) across the three breeds, while using information on pedigree relationships resulted in negligible differences, −0.0008% (±0.002). Furthermore, compared to the parental genome, the average (±SD) proportion of the genome in ROH of at least 5 Mb in the progeny using genomic data (i.e. **SNPRM** and **ROHRM**) decreased by 0.015% (±0.003) across the three breeds, while using pedigree relationships resulted in negligible differences, −0.002% (±0.004). Compared to the parental genome, negligible differences in average pedigree-based inbreeding were found when minimizing relationships using the **SNPRM** or **ROHRM**.

**Table 4:** Correlations of off-diagonal elements between different relationship matrices^a^ within each breed

Breed	Comparison	Correlation
LW	A, SNPRM	0.535
	A, ROHRM	0.665
	SNPRM, ROHRM	0.875
LA	A, SNPRM	0.630
	A, ROHRM	0.726
	SNPRM, ROHRM	0.91
DU	A, SNPRM	0.599
	A, ROHRM	0.726
	SNPRM, ROHRM	0.885

*DU* Duroc, *LW* Large White, *LA* Landrace

^a^ **A** refers to minimizing pedigree-based parent relationships; **SNPRM** refers to minimizing SNP-bySNP based parent relationships; **ROHRM** refers to minimizing ROH-based parent relationships

**Table 5:** Average (±SD) difference for multiple genome-wide diversity metrics between the parents and their associated progeny across different mate allocation scenarios

Breed	Diversity parameter^a^	Relationship used to constrain parental relationships^b^			
		Random	A	SNPRM	ROHRM
LW	Pedigree inbreeding	0.011 (0.001)	−0.001 (0.001)	0.004 (0.001)	0.004 (0.001)
	Heterozygosity	−0.007 (0.001)	−0.004 (0.001)	0.004 (0.001)	0.004 (0.001)
	ROH5 inbreeding	0.012 (0.002)	0.003 (0.002)	−0.012 (0.002)	−0.014 (0.002)
LA	Pedigree inbreeding	0.008 (0.001)	−0.003 (0.002)	0.001 (0.001)	0.001 (0.001)
	Heterozygosity	−0.003 (0.001)	0.001 (0.002)	0.006 (0.002)	0.005 (0.002)
	ROH5 inbreeding	0.006 (0.003)	−0.003 (0.003)	−0.013 (0.003)	−0.015 (0.003)
DU	Pedigree inbreeding	0.007 (0.001)	−0.007 (0.001)	−0.001 (0.001)	−0.002 (0.001)
	Heterozygosity	−0.003 (0.001)	0.001 (0.001)	0.008 (0.001)	0.006 (0.001)
	ROH5 inbreeding	0.003 (0.003)	−0.006 (0.002)	−0.017 (0.002)	−0.019 (0.002)

*DU* Duroc, *LW* Large White, *LA* Landrace

^a^Pedigree inbreeding: refers to diagonals of the pedigree-based relationship matrix; heterozygosity (%): proportion of SNPs that are heterozygous; ROH5 inbreeding (%): proportion of the genome that is in a ROH of at least 5 Mb

^b^Random refers to random mating; **A** refers to minimizing pedigree-based parent relationships; **SNPRM** refers to minimizing SNP-bySNP based parent relationships; **ROHRM** refers to minimizing ROH-based parent relationships

**Fig. 4:**
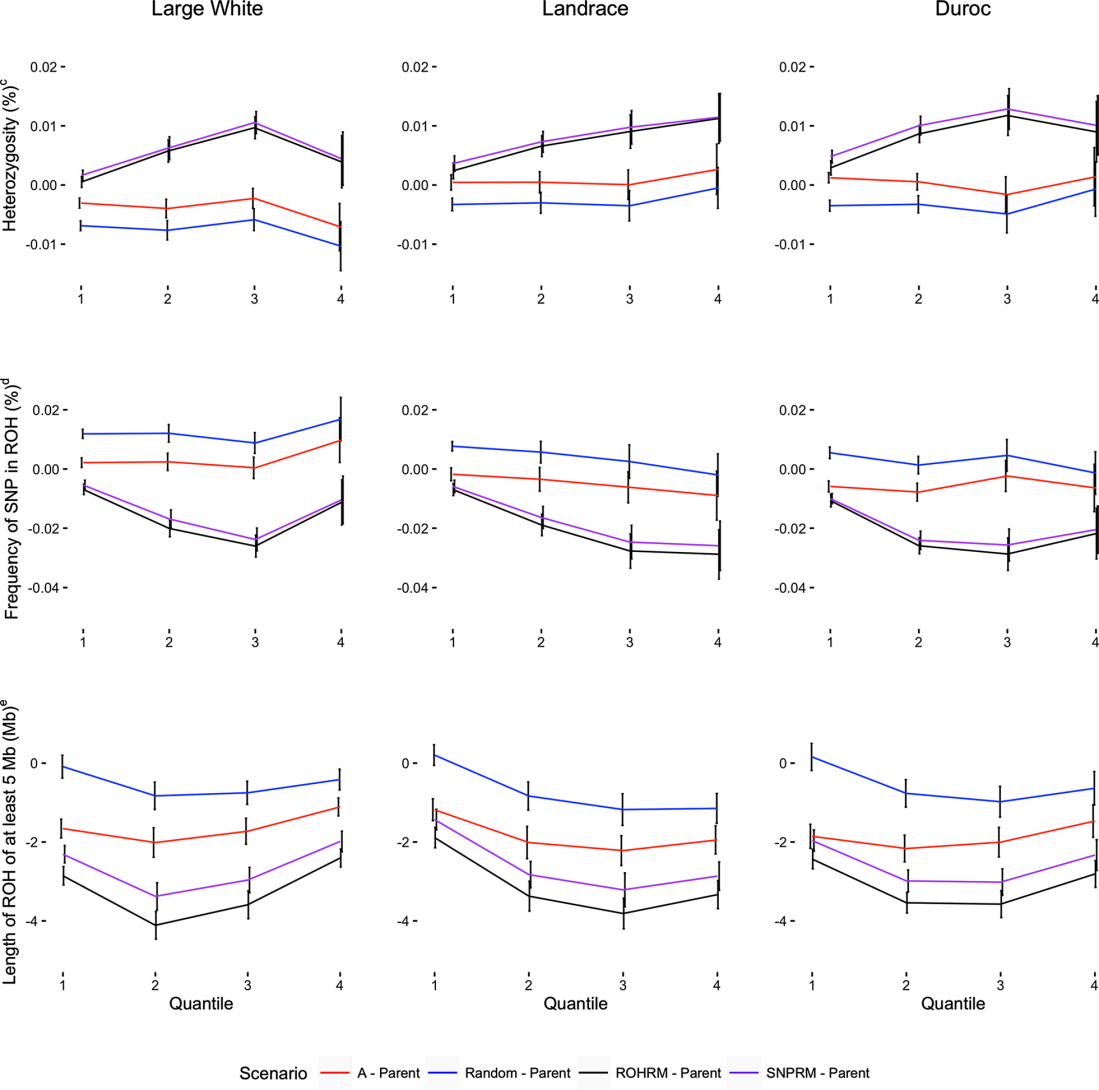
Average difference for multiple diversity metrics between the parents and their associated progeny per quantile^a^ across different mate allocation scenarios^b^. ^a^Quantile 1 was less than the 50th percentile; Quantile 2 was greater than or equal than 50th and less than the 75th percentile; Quantile3 was greater than or equal to the 75th and less than the 90th percentile; Quantile 4 was greater than or equal to the 90th percentile. ^b^Random refers to random mating; pedigree refers to minimizing pedigree based parent relationships; **SNPRM** refers to minimizing genomic-based parent relationships; **ROHRM** refers to minimizing run of homozygosity based parent relationships. ^c^Mean proportion of SNP heterozygous. ^d^Mean frequency of a SNP being in a run of homozygosity of at least 5 Mb. ^e^Mean ROH length (Mb) for SNP that were contained within an ROH of at least 5 Mb

To investigate the impact of minimizing parental relationships using either pedigree or genomic-based methods for regions with a low level of diversity, the genome was split into quantiles based on the frequency of ROH5. These results are shown in Fig. 4 as the difference between the progeny and parent genomes. A value of 0 implies no difference between the progeny and parental genomes. Across all breeds, the ability to differentially target low diversity regions, as quantified by either heterozygosity or ROH5, was achieved using either the **SNPRM** or **ROHRM** with similar effectiveness. For example, compared to the parental genome, the ROH5 frequency averaged (±SD) across breeds using genomic data (i.e. **SNPRM** and **ROHRM**) was reduced by 0.0076 (±0.003) and 0.0198% (±0.0106) for the progeny versus the parental generation for quantiles 1 (high level of genetic diversity) and 4 (low level of genetic diversity), respectively. In comparison, the frequency of ROH5 averaged (±SD) across breeds using **A** was reduced by 0.0019 (±0.003) and 0.0019% (±0.0115) for quantiles 1 and 4, respectively. Across quantiles, minimizing parental relationships based on pedigree did not target low diversity regions, as expected, due to the fact that common ancestors from multiple past generations contribute very little to variation in pedigree inbreeding, although it can contribute substantially to variation in the number and length of segments of the genome that are autozygous [11]. Furthermore, pedigree-based relationships are an expectation whereas **SNPRM** or **ROHRM** are more closely related to the realized relationship. Lastly, compared to the parental genome, constraining relationships based on the **ROHRM** resulted in the largest reduction in the length (Mb) of ROH that a SNP was in, although the **SNPRM** resulted in only a slightly smaller reduction than the **ROHRM**.

## Discussion

In this study, we characterized the frequency of ROH within purebred breeds and its persistence within the crossbred progeny. The availability of large genotyped multi-breed reference populations in swine breeding programs allows for a deeper dissection of the similarity between parental genomes that are used to breed the crossbred animals that are raised in a commercial setting. Furthermore, because the parents are routinely genotyped, we analyzed methods to manage the purebred genome and its impact on the crossbred genome. This methodology can be used as a tool to monitor diversity in the crossbred genome without having to genotype the crossbred individuals. This is of primary importance since breeding goals are defined to maximize crossbred performance and, thus, genome management methods should target the purebred level to achieve this.

### Genetic characterization of purebred and crossbred populations

Within each of the purebred populations that we analyzed, regions of the genome with a high frequency of medium (>5 Mb) and long (>10 Mb) ROH stretches occurred on the majority of the chromosomes. This result was as expected because all breeds have been closed to outside breeding stock for multiple generations and strongly selected; stretches of homozygosity are more frequent in selected versus unselected populations [44]. The purebred populations had similar levels of inbreeding based on multiple metrics. Furthermore, based on the PCA analysis, LA, LW and DU are divergent breeds, although the two maternal breeds (i.e. LA and LW) were more similar to each other compared to the terminal breed (DU). Multiple regions were found that had a high frequency of ROH across both the maternal breeds whereas one region was detected across all three breeds. The regions on SSC1 (248.7–264.2 Mb), SSC4 (42.1–61.3 Mb) and SSC14 (98.0–111.7 Mb) detected in LW and LA were previously shown to impact meat and carcass quality [48–51], multiple production and meat quality traits [52, 53], and reproduction [54], respectively. Four regions were found to have a higher frequency of ROH5 and/or ROH10 across the terminal DU breed and both the maternal breeds. The regions on SSC3 (36.4–59.5 Mb) and SSC14 (121.0–132.5 Mb) were found to be associated with meat and carcass quality traits [52, 55, 56], and with carcass quality [57] and fitness traits [58], respectively.

### Persistence of runs of homozygosity in maternal crossbred and commercial crossbred animals

The genomes of the crossbred dams and market animals were simulated from the observed parental genotypes and it was confirmed that haplotypes were shared between parental breeds. Furthermore, the crossbred dams displayed higher levels of shared haplotypes in the parental crosses than in the crossbred market animals. Persistence of ROH in crossbred animals indicates that these animals can be inbred for a portion of the genome, although their level of inbreeding based on pedigree information is zero. Identification of shared haplotypes also highlights the fact that portions of the genome have shared haplotypes across populations, which cannot be determined when the population is characterized at the genome-wide level. Previous work on a different population by Zanella et al. [8] also showed that shared haplotypes exist between the LW and LA breeds, although the frequency at which they occurred in the crossbred genome was not investigated. The LW breed originated in England as a cross between Cumberland, Leicecstershire, Middle and Small White breeds, with early registration records dating back to 1884 [59]. The LA breed was first derived in the late 1800 s as a cross between LW and a native Danish pig [59]. Both breeds have been bred as separate populations in many modern breeding programs to maximize commercial sow production through crossbreeding of these two breeds [59]. Previous work on human populations showed that multiple large (i.e. ≫1 Mb) ancestral haplotypes have persisted in outbred human populations and that these autozygous segments were more common in regions with low recombination rates and high linkage disequilibrium (LD) [60, 61]. Therefore, even if the relationship between the parents is distant, regions with low recombination rates (and therefore high LD) may have enabled the ancestral segment to persist intact across multiple generations, although this needs to be further investigated.

Persistence of ROH in crossbred animals results in decreased heterozygosity for that region, which reduces the degree of heterosis. As discussed previously, ROH have been shown to be enriched with deleterious variants, although the length at which the highest frequency of deleterious mutations occurs has been observed both in long ROH in human populations [22] and in short to medium ROH in cattle populations [21]. Previous research in dairy cattle showed that the additive effects of regions of high frequency of ROH for yield traits and/or calving interval have positive covariances, such that it is beneficial for the region to be in a long homozygous stretch [62]. Kim et al. [63] reported a similar result based on the regression of the most frequent haplotype on phenotype for multiple yield and fertility traits. Accumulation of inbreeding in parental lines is expected to result in an increase in the frequency of both favorable and deleterious haplotypes. Although deleterious haplotypes within regions that are under long-term directional selection may have been purged [21]. Given the high frequency of ROH in the parental populations within regions that persisted in the crossbred animals, it is hypothesized that these regions are most likely favorable haplotypes, although this needs to be confirmed with phenotypic data. However, genetic diversity will be low for regions with high frequencies of ROH. In general, persistence of long ROH stretches (i.e. >5 Mb) is detrimental. In the case of recent inbreeding, ROH stretches are likely to be enriched with deleterious mutations, which would result in reduced performance [21, 22]. Furthermore, a region with a reduced level of diversity compromises the chances to recruit new genetic variation due to linked polymorphisms being removed as the ROH increases in frequency [19]. This would be particularly relevant under fast changing environmental conditions. Lastly, the positive impacts of creating new favorable haplotype combinations by recombination are hampered in long stretches of ROH [20]. Managing purebred populations to maintain genetic diversity and reduce the length and frequency of ROH not only has desirable effects in terms of diversity at the nucleus level, but it also reduces the chance that long haplotypes are shared between breeds and allows for recombination to create new combinations of haplotypes in the crossbred animals.

### Mating designs to minimize long stretches of homozygosity within nucleus populations

Previous studies have used ROH as a metric to determine the population history of individuals across multiple groups [10]. The same measure could also be used to monitor the genome of a population as time proceeds in the form of the frequency and length of ROH that exist within the population. Prior to the advent of genomic information, populations were essentially managed to minimize accumulation of inbreeding at the genome-wide level either by constraining matings above a certain expected inbreeding cutoff [64] or using optimal contribution selection methods [23, 24]. With the advent of routine genotyping within swine breeding companies, novel methods can be used to manage inbreeding more precisely at the genomic level in nucleus populations. In the current study, minimizing relationships based on matrix **A** had an unfavorable effect on the diversity at the genomic level compared to both genomic metrics **SNPRM** or **ROHRM**. This is in agreement with previous simulation studies that spanned multiple generations in the context of conservation [65, 66] and livestock breeding programs [19, 25, 27, 30, 67]. Based on simulations, Sonesson et al. [25] found that the genomic rate of inbreeding was around 3 times higher when using optimal contributions constrained by pedigree versus genomic information. Similar results were reported by Pryce et al. [30] who used information from a SNP-by-SNP-based relationship metric similar to our study and showed an almost twofold reduction in inbreeding compared with using information from a pedigree-based relationship mating design. Rodríguez-Ramilo et al. [67] also found that genomic-based selection methods allowed more genetic diversity to be maintained in comparison to pedigree-based methods, as measured by observed heterozygosity.

Previous work in swine has shown that the correlation between pedigree and genomic kinships was slightly higher (r = 0.78) [68] than found here, although a similar correlation was reported for dairy cattle [69]. Furthermore, the correlation between the off-diagonal elements of **ROHRM** and **A** were similar to those previously reported for dairy cattle [30] and simulated data [35], for which a similar approach was used to construct the ROH-based relationship matrix. It should be noted that we did not assess the impact of using different relationship matrices in mating designs across multiple generations on the genetic diversity or ROH frequency across the genome. Future studies using simulation and/or real data should investigate the long-term effect of minimizing different relationship metrics and its impact on fitness of the population. Furthermore, we found no difference between **SNPRM** and **ROHRM** across quantiles for heterozygosity or ROH5 (Fig. 4). Previous simulations, based on multiple generations showed that **SNPRM** maintains heterozygosity to a greater degree than **ROHRM** [66, 67], which is not surprising since the **SNPRM** is more closely related to the heterozygosity than the **ROHRM**. Within one generation, the level of heterozygosity was numerically the highest when the **SNPRM** was used for the majority of the quantiles across breeds compared to the **ROHRM**, which is in line with previous work, although the across-replicate standard deviation covered both means.

To the best of our knowledge, the effect of different mate allocation strategies on the length of ROH has not been investigated. The **ROHRM** reduced the length of ROH to a greater degree than the **SNPRM**, which has implications for reducing regions of low genetic diversity and breaking down long haplotypes in the parental lines, thus reducing the occurrence of long stretches persisting in the crossbred offspring. Using a related approach based on integrated haplotype homozygosity score, Bosse et al. [66] found that a relationship matrix similar to the **ROHRM** was more efficient in reducing the presence of long similar haplotypes in the next generation than a matrix that was similar to the **SNPRM** used here. Recent work by Gómez-Romano et al. [27] also investigated methods to maintain genetic diversity in certain regions based on optimal contribution theory. They obtained similar results, i.e. that diversity was maintained at pre-defined regions by constraining genomic relationships for that region. However, to prevent a substantial increase in the rate of coancestry across the rest of the genome, an additional constraint based on genomic relationships across the rest of the genome had to be applied. An alternative approach could be to design mating programs at the crossbred level (i.e. parents of crossbred progeny) to maximize heterozygosity in the crossbred, instead of at the nucleus level. We did not investigate this approach due to a lack of genotypes for individuals in the multiplier tier of the breeding pyramid. However, as cheap genotyping strategies and reproductive technologies get introduced this may become possible, as outlined by Visscher et al. [70].

Limitations of the current study involve the generation of the ROH5 and ROH10 statistics. Only ROH cutoff values of 5 and 10 were used because the medium density SNP panel that was used was not sensitive enough for accurate determination of short ROH segments [71]. Furthermore, because of the uneven distribution of SNPs across the genome, multiple editing procedures were used to limit the number of spurious regions with a high frequency of ROH. The use of these strict editing procedures may have resulted in regions of the genome with a high frequency of ROH to be missed, although this will become less important as the density of SNP genotyping platforms increases. Another limitation in the simulation to create crossbred genotypes is our implicit assumption that the animals in the nucleus generate the crossbred dams used in commercial farms. In a traditional swine breeding program, a multiplier and/or daughter nucleus uses the genetic material of the nucleus animals to generate crossbred dams, which are used on the commercial farms. Due to this, more than one generation of gamete creation and recombination events will occur between the nucleus and the actual generation of crossbred dams and therefore may result in a lower frequency and shorter length of ROH than what is observed in the simulation. Actual crossbred genotypes were not available to confirm the results generated from the simulation.

## Conclusions

Regions of high frequencies of ROH5 and/or ROH10 across at least two breeds were detected on SSC1, 4, 9 and 14. More importantly, ROH in the parental breeds were shown to persist in the crossbred dams and, to a lesser degree, in market animals via shared haplotypes in the parental breeds. This has implications for the level of heterozygosity at the crossbred level. We also showed that it is possible to differentially target low diversity regions within the genome of purebred animals, as quantified by either heterozygosity or ROH5, by using either the **SNPRM** or **ROHRM**. We also identified differences in how effective different relationship measures were at reducing the length of a ROH across the majority of the ROH5-based quantiles across three breeds, with the **ROHRM** achieving the greatest reduction in ROH lengths. Finally, use of pedigree-based relationships in mating programs resulted in negligible changes in comparison to the changes observed when minimizing relationships based on genomic information. In conclusion, managing the genome at the nucleus level has positive impacts on maintaining the genetic diversity and decreasing the length and frequency of ROH at the nucleus level.

## Additional files

**Additional file 1.** C++ code for creating run of homozygosity (ROH) based relationship matrices. This file contains the C++ code to generate a ROH-based relationship matrix along with a tutorial of how to compile and run the program.

**Additional file 2.** Test_Genotype. This file contains phased genotype information in order to provide an example of how to run the program outlined in Additional file 1. Column one is the animal ID and column 2 is the phased genotype information.

**Additional file 3.** Test_Map. This file contains map information that relates to the Test_Genotype file (i.e. Additional file 2) in order to provide an example of how to run the program outlined in Additional file 1. Column one is the chromosome number and column 2 is nucleotide position.

**Additional file 4.** Previously detected QTL based on AnimalQTLdb for regions with high levels of ROH within purebred populations. Description of QTL associations as outlined in AnimalQTLdb for regions with high levels of ROH within purebred populations.
