# Supplementary material for: Characterization and management of long runs of homozygosity in parental nucleus lines and their associated crossbred progeny
Source: Genet Sel Evol. 2016 Nov 24;48:91. doi: 10.1186/s12711-016-0269-y (PMC5123398; doi:10.1186/s12711-016-0269-y)
Supplement: Supplementary file 1 — Additional file 1. C++ code for creating run of homozygosity (ROH) based relationship matrices. This file contains the C++ code to generate a ROH-based relationship matrix along with a tutorial of how to compile and run the program. [file 12711_2016_269_MOESM1_ESM.docx]

**Additional file 1**

**C++ code for creating run of homozygosity (ROH) based relationship matrices**

The following parameters in the given order are needed for the program to run:

**1.) Map File:** A map file with chromosome and position in Megabases (Mb) separated by a space. The map file name has to be composed of one word. An example is provided (“Test_Map”).

**2.) Genotype File:** A file that contains the animal ID and phased genotype data that is in a continuous string. The delimiter between the two variables is a space. Their can be no missing genotypes and the genotypes are coded as 0 (a_1_a_1_), 2(a_2_a_2_), 3(a_1_a_2_) and 4 (a_2_a_1_). The length of the string should be equal to the number of rows as the map file. The genotype file name has to be composed of one word and an example is provided (“Test_Genotype”).

**3.) ROH Mb cutoff:** The cutoff length in Mb of the window size for each ROH. For example a cutoff length of 5 Mb means that all windows have to span at least 5 Mb and the window is increased by one SNP until the criterion is satisfied.

**4.) ROH SNP Number Threshold:** The minimum number of standard deviations away from the mean number of SNPs within an ROH window that can be included in the analysis. For instance, a value of 2 represents any ROH window that has less than 2 times the average window count and will not be used to construct the ROH relationship matrix.

**5.) Form of Output:** Determines whether the file should be written in triplet form or matrix form. Triplet form is referred to by a 0 and matrix form by any other number.

Once the program is completed a ROH-based relationship will be written to the directory it is run in.

The code can be compiled on any mac or linux system by typing at terminal command line:

g++ ROHRM_Creator.cpp –o ROH_Relationship

Using the example files that give the program can be run by calling:

./ROH_Relationship Test_Map Test_Genotype 3000000 4 0

Any questions or problems compiling or running the code can be sent to jthoward@ncsu.edu.

The Source_Code is provided below, a separate file containing the code is provided as ROHRM_Creator.cpp:

#include <iostream>

#include <fstream>

#include <cstring>

#include <cstdlib>

#include <string>

#include <vector>

#include <cmath>

#include <ctime>

/**********************************************************/

/* ROH Index Object (Keeps track of ROH Windows) */

/**********************************************************/

class ROH_Index

{

private:

int Chromosome; /* Which Chromosome belongs to */

int StartPosition; /* Start position (Mb) of roh window */

int EndPosition; /* End position (Mb) of roh window */

int StartIndex; /* Start position (column number) of roh window */

int EndIndex; /* End position (column number) of roh window */

int NumberSNP; /* Number of SNP in ROH Window */

public:

// Constructors

ROH_Index();

ROH_Index(int chr = 0, int stpos = 0, int enpos = 0, int stind = 0, int enind = 0, int numsnp = 0);

// Destructors

~ROH_Index();

// Functions to grab parts of object

int getChr(){return Chromosome;}

int getStPos(){return StartPosition;}

int getEnPos(){return EndPosition;}

int getStInd(){return StartIndex;}

int getEnInd(){return EndIndex;}

int getNumSNP(){return NumberSNP;}

};

// Constructors ROH_Index

ROH_Index::ROH_Index(){Chromosome = 0; StartPosition = 0; EndPosition = 0; StartIndex = 0; EndIndex = 0; NumberSNP = 0;}

ROH_Index::ROH_Index(int chr, int stpos, int enpos, int stind, int enind, int numsnp)

{

Chromosome = chr; StartPosition = stpos; EndPosition = enpos; StartIndex = stind; EndIndex = enind; NumberSNP = numsnp;

}

// Destructors

ROH_Index::~ROH_Index(){}

using namespace std;

int main(int argc, char* argv[])

{

cout<<"╦═╗╔═╗╦ ╦╦═╗╔╦╗ ╔═╗┬─┐┌─┐┌─┐┌┬┐┌─┐┬─┐ \n";

cout<<"╠╦╝║ ║╠═╣╠╦╝║║║ ║ ├┬┘├┤ ├─┤ │ │ │├┬┘ \n";

cout<<"╩╚═╚═╝╩ ╩╩╚═╩ ╩────╚═╝┴└─└─┘┴ ┴ ┴ └─┘┴└─ \n";

cout<<"+--------------+-------------+----------------------------------------+"<<endl;

cout<<"|ROH relationship creator |\n";

cout<<"|Authors: Jeremy T. Howard (jthoward@ncsu.edu) |\n";

cout<<"|Institution: NCSU |\n";

cout<<"|Date: 02/01/2016 |\n";

cout<<"|This program is free software: you can redistribute it and/or modify |\n";

cout<<"|it under the terms of the GNU General Public License as published by |\n";

cout<<"|the Free Software Foundation, either version 3 of the License, or |\n";

cout<<"|(at your option) any later version. |\n";

cout<<"+--------------+-------------+----------------------------------------+"<<endl;

cout << "==================================================\n";

cout << "==\tParameters Specified \t==\n";

cout << "==================================================\n"<<endl;

if(argc != 6){cout << "Wrong nummber of parameters given" << endl; exit (EXIT_FAILURE);}

cout << " - Map File: " << argv[1] << endl;

cout << " - Genotype File: " << argv[2] << endl;

cout << " - ROH Mb Cutoff: " << argv[3] << endl;

cout << " - ROH Threshold: " << argv[4] << endl;

cout << " - Output format: " << argv[5] << endl;

/* Map File: Chromosome Mb position with a space dilemeter */

string mapfile = argv[1];

/* Genotype File: ID then string of genotypes */

string genofile = argv[2];

/* Mb cutoff for ROH */

int roh_cutoff = atoi(argv[3]);

/* Number times the mean to cutoff if too low of SNP in ROH */

int roh_threshold = atoi(argv[4]);

/* Output format hlaf/stored triplet or regular */

int form = atoi(argv[5]);

cout << "==================================================\n";

cout << "==\tReading in Map Genotype file \t==\n";

cout << "==================================================\n"<<endl;

/* Read in map file don't need to know how many SNP are in the file */

vector <string> numbers;

string line;

/* Import file and put each row into a vector then grab chromsome and position */

ifstream infile;

infile.open(mapfile.c_str());

while (getline(infile,line)){numbers.push_back(line);}

/* Total number of SNP */

int rows = numbers.size();

cout << " - Total Number of SNP in Map file is " << rows << endl;

/* stores chromosome in vector */

vector < int > chr(rows,0);

/* stores position in Mb */

vector < int > positionMb(rows,0);

/* position SNP is referring to in genotype string it is on */

int index[rows];

for(int i = 0; i < numbers.size(); i++)

{

/* grab chromosome number */

string temp = numbers[i];

size_t pos = temp.find(" ", 0);

string tempa = temp.substr(0,pos);

chr[i] = atoi(tempa.c_str());

/* grab position Mb */

temp.erase(0, pos+1);

positionMb[i] = atoi(temp.c_str());

/* Save index */

index[i] = i;

}

numbers.clear();

cout << "==================================================\n";

cout << "==\tIndexing Positions for ROH \t==\n";

cout << "==================================================\n"<<endl;

/* Fill ROH Index Object with each ROH window */

vector < ROH_Index > roh_index;

/* Create index to grab correct columns from genotype file when constructing ROH and Autozygosity */

for(int i = 0; i < rows; i++)

{

int diff[rows];

for(int j = 0; j < rows; j++)

{

/* if within the same chromosome then can be in an roh */

if(chr[i] == chr[j]){diff[j] = positionMb[j] - positionMb[i];}

/* if in another chromosome give it a 0 */

if(chr[i] != chr[j]){diff[j] = 0;}

}

/* determine where to grab */

int j = 0;

while(j < rows)

{

/* Determine where first one that is over threshold is reached */

if(diff[j] > roh_cutoff){break;}

j++;

}

/* Tabulate number of SNP in roh window */

int numsnp = index[j] - index[i] + 1;

if(j < rows)

{

/* fill ROH index with the window */

ROH_Index roh_temp(chr[i],positionMb[i],positionMb[j],index[i],index[j],numsnp);

roh_index.push_back(roh_temp); /* store in vector of roh_index objects */

}

}

cout << " - Total number of ROH windows prior to removal " << roh_index.size() << endl;

/* Figure out mean and Standard Deviation of number of SNP within ROH windows */

double sum = 0.0;

for(int i = 0; i < roh_index.size(); i++){sum += roh_index[i].getNumSNP();}

double mean = sum / roh_index.size();

double sq_sum;

for(int i = 0; i < roh_index.size(); i++){sq_sum += roh_index[i].getNumSNP() * roh_index[i].getNumSNP();}

double stdev = sqrt(sq_sum / roh_index.size() - mean * mean);

cout << " - Mean +/- S.D. SNP size for an ROH: " << mean << " " << stdev << endl;

int SNPSizeCutoff = mean - roh_threshold * stdev + 0.5; /* Ensures it rounds up */

cout << " - Any ROH window with SNP size below: " << SNPSizeCutoff << " removed." << endl;

/* Remove ROH windows that fall below threshold */

int row = roh_index.size(); /* Figure out number of rows */

int i = 0; /* counter to determine where you are at */

/* Keep ROH that are greater than a given threshold */

while(i < row)

{

while(1)

{

if(roh_index[i].getNumSNP() < SNPSizeCutoff)

{

roh_index.erase(roh_index.begin()+i); /* Remove from allfreq */

row = row - 1; /* One less column in G and row in allfreq */

break;

}

else

{

i++;

break;

}

}

}

cout << " - Total number of ROH windows after removal " << roh_index.size() << endl;

cout << "==================================================\n";

cout << "==\tReading in Genotype file \t==\n";

cout << "==================================================\n"<<endl;

/* Stores Individual ID */

vector < string > ID;

/* Stores genotypes as a string */

vector < string > geno;

/* Read in Genotype file */

ifstream infile1;

infile1.open(genofile.c_str());

while (getline(infile1,line))

{

/* Grab Individual ID */

size_t pos = line.find(" ",0);

string tempa = line.substr(0,pos);

ID.push_back(tempa);

line.erase(0,pos+1);

/* Grab Genotype string */

geno.push_back(line);

}

cout << " - Number of animals with genotypes: " << geno.size() << "." << endl;

cout << " - Number of Genotypes per animal: " << geno[0].size() << "." << endl;

cout << "==================================================\n";

cout << "==\tConstructing ROH Relationship Matrix \t==\n";

cout << "==================================================\n"<<endl;

/* store relationship values in 2-D vector and build first and initialize to 0.0 */

vector < vector < double > > ROH_Relationship;

for(int i = 0; i < geno.size(); i++)

{

vector < double > temp;

for(int j = 0; j < geno.size(); j++){temp.push_back(0.0);}

ROH_Relationship.push_back(temp);

}

cout << " - ROH Relationship Matrix (Size = " << ROH_Relationship.size() << " x " << ROH_Relationship[0].size() << ")." << endl;

/* Loop across all ROH Windows */

for(int r = 0; r < roh_index.size(); r++)

{

/* First step is to get number of unique haplotypes to set dimension of Haplotype similarity matrix (H) */

/* stores each unique ROH roh */

vector < string > haplotypes;

/* Index for paternal haplotype number of a given animal */

vector < int > AnimalPatHap;

/* Index for maternal haplotype number of a given animal */

vector < int > AnimalMatHap;

/* loop across animals */

for(int i = 0; i < geno.size(); i++)

{

/* Grab specific haplotype */

string temp = geno[i].substr(roh_index[r].getStInd(),roh_index[r].getNumSNP());

/* First initalize homo1 and homo2 as genotypes then convert them to phased haplotypes */

string homo1 = temp; /* Paternal haplotypes */

string homo2 = temp; /* Maternal haplotypes */

for(int g = 0; g < temp.size(); g++)

{

if(homo1[g] == '0'){homo1[g] = '1';} /* a1a1 genotype */

if(homo2[g] == '0'){homo2[g] = '1';} /* a1a1 genotype */

if(homo1[g] == '2'){homo1[g] = '2';} /* a2a2 genotype */

if(homo2[g] == '2'){homo2[g] = '2';} /* a2a2 genotype */

if(homo1[g] == '3'){homo1[g] = '1';} /* a1a2 genotype */

if(homo2[g] == '3'){homo2[g] = '2';} /* a1a2 genotype */

if(homo1[g] == '4'){homo1[g] = '2';} /* a2a1 genotype */

if(homo2[g] == '4'){homo2[g] = '1';} /* a2a1 genotype */

}

/* Loop across two gametes and see if unique if so put in haplotype library */

for(int g = 0; g < 2; g++)

{

string temp;

if(g == 0){temp = homo1;}

if(g == 1){temp = homo2;}

/* Haplotype library will be empty for first individual */

if(haplotypes.size() == 0){haplotypes.push_back(temp);}

/* has to not match up with all unique haplotypes before added */

int num = 0;

if(haplotypes.size() > 0)

{

for(int h = 0; h < haplotypes.size(); h++)

{

/* Doesn't equal keep going until reach end then add */

if(temp.compare(haplotypes[h]) != 0){num++;}

/* if does equal save index */

if(temp.compare(haplotypes[h]) == 0 && g == 0){AnimalPatHap.push_back(h);}

/* if does equal save index */

if(temp.compare(haplotypes[h]) == 0 && g == 1){AnimalMatHap.push_back(h);}

}

}

if(num == haplotypes.size())

{

/* If number not match = size of hapLibary then add */

haplotypes.push_back(temp);

/* save to index later */

if(g == 0){AnimalPatHap.push_back(haplotypes.size()-1);}

/* save to index later */

if(g == 1){AnimalMatHap.push_back(haplotypes.size()-1);}

}

} /* Close loop that loops through twice, once for each gamete */

}

/* all unique haplotypes are tabulated now create Haplotype similarity matrix (H) */

/* store in 2-D vector to not store in heap instead of stack */

vector < vector < double > > H_Matrix;

/* Build 2-D vector and fill off-diagonals as 0.0 and diagonals as 1.0 */

for(int i = 0; i < haplotypes.size(); i++)

{

vector < double > temp;

for(int j = 0; j < haplotypes.size(); j++)

{

if(i == j){temp.push_back(1.0);}

if(i != j){temp.push_back(0.0);}

}

H_Matrix.push_back(temp);

}

/* Haplotype similarity matrix (H) created now construct add result to ROH_Relationship Matrix */

/* This results in an animal being related only if it has the exact same haplotype, if not the */

/* exact same assume unrelated */

for(int ind1 = 0; ind1 < AnimalPatHap.size(); ind1++)

{

for(int ind2 = ind1; ind2 < AnimalPatHap.size(); ind2++)

{

ROH_Relationship[ind1][ind2] += (H_Matrix[AnimalPatHap[ind1]][AnimalPatHap[ind2]] +

H_Matrix[AnimalPatHap[ind1]][AnimalMatHap[ind2]] +

H_Matrix[AnimalMatHap[ind1]][AnimalPatHap[ind2]] +

H_Matrix[AnimalMatHap[ind1]][AnimalMatHap[ind2]]) / 2;

ROH_Relationship[ind2][ind1] = ROH_Relationship[ind1][ind2];

} /* Finish loop across ind2 */

} /* Finish loop across ind1 */

if(r % 1000 == 0){cout << endl << " - " << r;}

}

cout << endl;

/* Once finished computing the relationship matrix now divide by total number of ROH windows */

for(int ind1 = 0; ind1 < geno.size(); ind1++)

{

for(int ind2 = ind1; ind2 < geno.size(); ind2++)

{

ROH_Relationship[ind1][ind2] = ROH_Relationship[ind1][ind2] / roh_index.size();

ROH_Relationship[ind2][ind1] = ROH_Relationship[ind1][ind2];

} /* Finish loop across ind2 */

} /* Finish loop across ind1 */

/* Output Matrix to file */

fstream checkRel; checkRel.open("ROH_Relationship_Matrix", std::fstream::out | std::fstream::trunc); checkRel.close();

std::ofstream output9("ROH_Relationship_Matrix", std::ios_base::app | std::ios_base::out);

if(form==0){

for(int ind1 = 0; ind1 < geno.size(); ind1++)

{

for(int ind2 = 0; ind2 < geno.size(); ind2++)

{

if(ind2 >= ind1){

output9 << ind1+1 << " "<<ind2+1<<" ";

output9 << ROH_Relationship[ind1][ind2];

output9<<endl;

}

} /* Finish loop across ind2 */

} /* Finish loop across ind1 */

cout << " - Finished Constructing ROH Relationship Matrix (Size = " << geno.size() << " x " << geno.size() << ")." << endl;

}

if(form!=0){

for(int ind1 = 0; ind1 < geno.size(); ind1++)

{

for(int ind2 = 0; ind2 < geno.size(); ind2++)

{

if(ind2 != geno.size() - 1){output9 << ROH_Relationship[ind1][ind2] << " ";}

if(ind2 == geno.size() - 1){output9 << ROH_Relationship[ind1][ind2];}

} /* Finish loop across ind2 */

output9 << endl;

} /* Finish loop across ind1 */

cout << " - Finished Constructing ROH Relationship Matrix (Size = " << geno.size() << " x " << geno.size() << ")." << endl;

}

}
